# Near-Infrared Photoelectric Properties of Multilayer Bi_2_O_2_Se Nanofilms

**DOI:** 10.1186/s11671-019-3179-4

**Published:** 2019-12-09

**Authors:** Hang Yang, Wei Chen, Xiaoming Zheng, Dongsheng Yang, Yuze Hu, Xiangzhe Zhang, Xin Ye, Yi Zhang, Tian Jiang, Gang Peng, Xueao Zhang, Renyan Zhang, Chuyun Deng, Shiqiao Qin

**Affiliations:** 1 College of Arts and Science, National University of Defense Technology, Changsha, 410073 China; 2 College of Advanced Interdisciplinary Studies, National University of Defense Technology, Changsha, 410073 China; 3 Department of Chemistry, National University of Singapore, Singapore, 117543 Singapore; 4 College of Physical Science and Technology, Xiamen University, Xiamen, 361005 China

**Keywords:** Bi_2_O_2_Se, Multilayer, Photodetector, Near-Infrared

## Abstract

The near-infrared (NIR) photoelectric properties of multilayer Bi_2_O_2_Se nanofilms were systematically studied in this paper. Multilayer Bi_2_O_2_Se nanofilms demonstrate a sensitive photo response to NIR, including a high photoresponsivity (~ 101 A/W), a quick response time (~ 30 ms), a high external quantum efficiency (~ 20,300%), and a high detection rate (1.9 × 10^10^ Jones). These results show that the device based on multilayer Bi_2_O_2_Se nanofilms might have great potentials for future applications in ultrafast, highly sensitive NIR optoelectronic devices.

## Background

Infrared (IR) photodetectors have been widely investigated and studied since their delicate applications in military, commercial, public, and academic domains [1–3]. In the past decade, two-dimensional (2D) materials, for example, graphene, transition metal dichalcogenides (TMDs), and black phosphorus, have grown as promising candidates with great potential for infrared applications [4–9]. Due to the intriguing properties of 2D materials, including the ultrathin thickness, highly mechanical flexibility, suitable and tunable band gap, ultrafast optoelectronic characteristics, and easily tailored van der Waals heterostructures, 2D layered materials have been considered the competitive IR media for next-generation photodetectors [10–12].

Very recently, layered bismuth oxyselenide (Bi_2_O_2_Se) was discovered as a promising 2D semiconductor with high electron mobility, ultrafast photoresponse, excellent environmental stability, and easy-accessibility to large production via a facile chemical vapor deposition (CVD) method, making it attractive for electronic and optoelectronic applications [7, 8, 13–15]. Previously, He Jun et al. [7] and Peng Hailin et al. [8] successively reported that Bi_2_O_2_Se owned excellent photoelectric properties to near-infrared (NIR). However, they mainly concerned about thin-layer Bi_2_O_2_Se (thickness ~ 7 nm). Prior studies with respect to other 2D materials, such as MoS_2_ [16] and MoSe_2_ [17, 18], showed multilayer nanoflakes also owned an extraordinary photoelectric performance compared with monolayer or thin-layer. In fact, multilayer Bi_2_O_2_Se may be more attractive than thin-layer Bi_2_O_2_Se for FET applications in the thin-film transistor (TFT) configuration [16, 19]. For example, the density of states in multilayer Bi_2_O_2_Se is much higher than that in thin-layer Bi_2_O_2_Se, which can produce considerably high drive currents in the ballistic limit [13, 14]. In long-channel TFTs, multiple conducting channels can be created by field-effects in multilayer Bi_2_O_2_Se, which can boost the current drive of TFTs, similar to silicon-on-insulator MOSFETs [19]. Moreover, multilayer Bi_2_O_2_Se offers a wider spectral response than thin-layer Bi_2_O_2_Se, due to its narrower bandgap, which can be advantageous in a variety of photodetector applications [20]. Yet, multilayer Bi_2_O_2_Se–based photodetectors have not been extensively studied for use in electronics or optoelectronics.

Therefore, the NIR photoelectric properties of multilayer Bi_2_O_2_Se (thickness ~ 30 nm) were systematically studied in this paper. Multilayer Bi_2_O_2_Se–based photodetector demonstrates an ultra-sensitive photoresponse from 850 to 1550 nm with a good reproducibility at room temperature. Its photoresponsivity reaches 101 A/W at 1000 nm, along with a fast rise time and a decay time of 30 ms and 60 ms, respectively. Compared with thin-layer Bi_2_O_2_Se, multilayer Bi_2_O_2_Se has higher photoresponsivity and external quantum efficiency, while still keeps a relatively fast response time and a high detection rate. In addition, the photocurrent exhibits a linear dependence on the incident power, offering a good tune ability for multi-purpose applications. These results offer the opportunities for developing the next generation of ultra-sensitive high-performance NIR room-temperature photodetectors.

## Methods

### Growth and Characterization of Bi_2_O_2_Se

The Bi_2_O_2_Se nanofilms were synthesized via a chemical vapor deposition (CVD) method. Bi_2_O_3_ and Bi_2_Se_3_ (Alfa Aesar) were located at the center of the horizontal tubular furnace (Lindberg/Blue M), and the mica substrates (Tiancheng Fluorphlogopite Mica Company Ltd., China) were placed downstream as substrates. The furnace was firstly heated to 640 °C with elevation rate of 30 °C min^−1^ and kept for 60 min with an argon gas flow. Finally the furnace was cooled down to room temperature naturally. The synthesized samples were characterized by optic microscope (Olympus BX51), Raman spectrum (WiTec 300R), atomic force microscope (semi-contact mode, NT-MDT company) scanning electron microscope (FEI company). Here, 10-nm aluminum was firstly thermal evaporated to avoid charge effect of mica substrate before SEM characterization.

### Device Fabrication

The photodetector based on multilayer Bi_2_O_2_Se was fabricated by a standard micro-nano technology. The source and drain contacts were defined by e-beam lithography and followed by depositing a 5 nm Cr/50 nm Au metal stack applying e-beam evaporation. Note that, in order to prevent the charge accumulation on mica substrate during EBL process, conductive polymer photoresist (SX AR-PC-5000) was spin coated on mica prior to the EBL process. Finally, the device was bonded on the chip carrier for further photoelectric measurement.

### Performance Measurement

The photocurrent measurements were performed by a homemade xenon lamp (light source: BETICAL HDL-II) photo-detection platform. In the measurement, Keithley 2450 was used to supply the source–drain bias. By switching on/off the light, the drain currents at on/off states were collected. The photoelectric response of the device at different wavelengths (850–1550 nm) could be obtained by substituting different filters.

## Results and Discussion

**Fig. 1:**
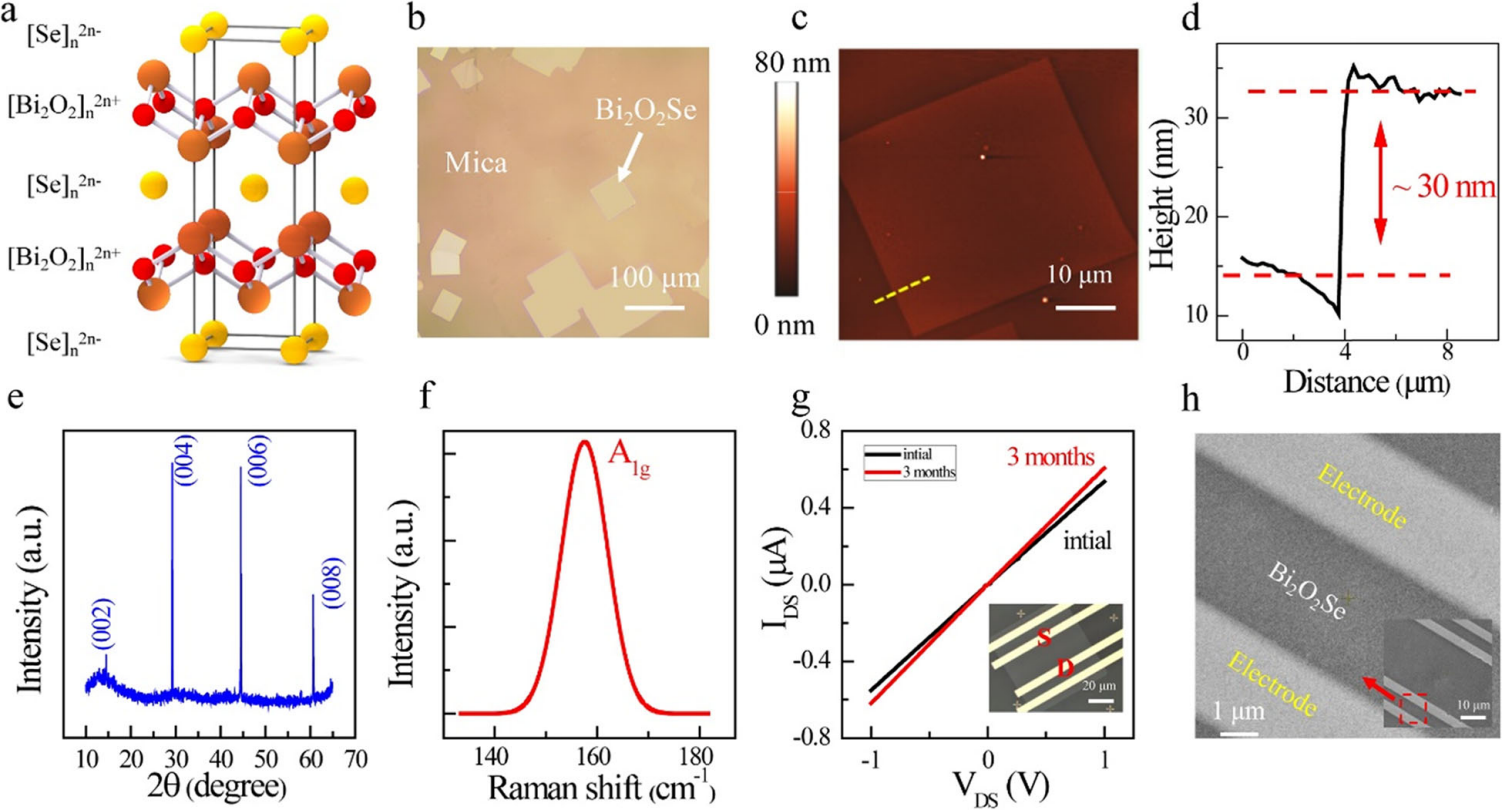
Characterization of layered Bi_2_O_2_Se nanofilms. **a** Schematic of layered Bi_2_O_2_Se crystal structure. Orange ball: Bi. Red ball: O. Yellow ball: Se. **b** Typical optical image of as-grown Bi_2_O_2_Se nanofilms on mica. **c** AFM image of multilayer Bi_2_O_2_Se nanofilms. **d** Corresponding height information. The thickness is ~ 30 nm. **e** XRD patterns. **f** Raman spectrum excited using a laser of 532 nm. **g** Output characteristics of multilayer Bi_2_O_2_Se device, showing an excellent environmental stability even exposed to air for 3 months. The inset shows the optic image of the device. **h** SEM image of multilayer Bi_2_O_2_Se nanofilms, showing the nanostructure information of this material. The inset is a magnified SEM image

As Fig. 1a illustrates, layered Bi_2_O_2_Se shows a tetragonal structure with I4/mmm space group, and consists of planar covalently bonded oxide layers (Bi_2_O_2_) sandwiched by Se square arrays with relatively weak electrostatic interactions [21]. This kind of structure is similar to mica. Hence, two-dimensional Bi_2_O_2_Se nanofilms are all synthesized on the mica substrate by chemical vapor deposition (CVD) method so far [7, 14, 15]. Figure 1b illustrates a large areal optical view of as-grown multilayer Bi_2_O_2_Se nanofilms on mica. It is clearly seen that the nanofilms are uniform and almost present rectangular shapes. An atomic force microscope (AFM) images of Bi_2_O_2_Se nanofilms in our experiment are shown in Fig. 1c. According to the theoretical thickness of monolayer (≈ 0.61 nm) [14, 15], 30 nm (Fig. 1d) equals the thickness of around 49 layers. Figure 1e shows the XRD patterns of Bi_2_O_2_Se nanofilms. Discernable peaks are all attributed to (00l) diffraction planes of Bi_2_O_2_Se (the crystalline orientation is along *c*-axis), consistent with the previous studies [14]. The characteristic A_1g_ peak of Bi_2_O_2_Se can be found at ≈ 159.1 cm^−1^ in the Raman spectrum (Fig. 1f), which is in good consistency with the prior reports [22]. Figure 1g shows the typical I-V curve of Bi_2_O_2_Se device. Excellent linear I-V curve indicates the Ohmic contacts are formed. Besides, 2D Bi_2_O_2_Se-based photodetector demonstrates an excellent environmental stability, which is a key metric for future practical applications [14, 15]. From Fig. 1h, the measured length and width of the device is 29 μm and 91 μm, respectively.

**Fig. 2:**
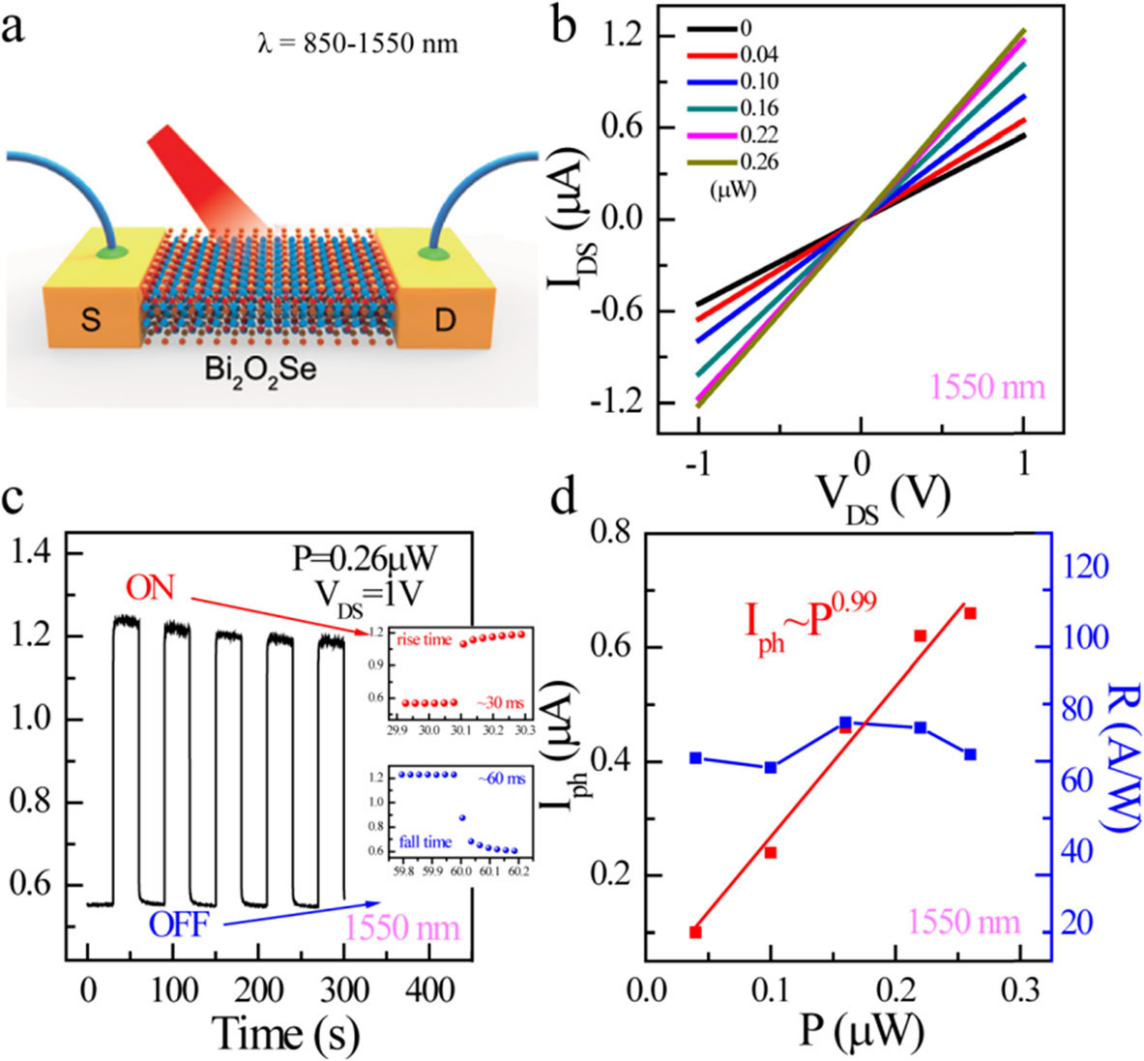
Photoelectric response to telecommunication band (wavelength 1550 nm) of multilayer Bi_2_O_2_Se–based photodetector. **a** Schematic 3D view of Bi_2_O_2_Se-based photodetector under illumination. **b** I-V curves of Bi_2_O_2_Se-based photodetector under different light intensities. **c** Time-dependent photoresponse behaviors of Bi_2_O_2_Se device under 1550-nm light illumination (*P* = 0.26 uW). The inset shows an ultrafast photo response of the device. **d** Photocurrent and photoresponsivity of Bi_2_O_2_Se-based photodetector under different light intensities

As Fig. 2a shows, the photoelectric response of multilayer Bi_2_O_2_Se–based photodetector to NIR was deliberately measured. Here, we mainly discuss the performance of the device in the telecommunication band (1550 nm), which is widely applied in military, commercial, public, and academic domains. It can be seen from Fig. 2b that I_DS_ obviously grows as the light intensity increases. Moreover, the I-V curve of the device under illumination does not demonstrate apparent open circuit voltage and short circuit current. This fact indicates that the Schottky barrier formed between the electrode and the material does not play a pivotal role in the transport characteristic of the device. Therefore, the photoelectric response of the material should mainly come from the photoconductive effect [10].

To evaluate the performance of the photodetectors, photoresponsivity (*R*), external quantum efficiency (*η*), and detectivity (D*) are critical parameters which can be calculated by the following formula [10, 17]:

1 $$ R={I}_{ph}/ PS $$

2 $$ \eta \left(\lambda \right)={R}_{\lambda } hc/ q\lambda $$

3 $$ {D}^{\ast }={I}_{ph}/P{\left(2 qS{I}_d\right)}^{1/2} $$

where *I*_*ph*_ is photocurrent (the difference of the drain current between illuminated (*I*_*i*_) and dark (*I*_*d*_) states), *P* is the light intensity, *S* is the effective area, *h* is the Planck’s constant, *c* is the light velocity, *λ* is the light wavelength, and *q* the electronic charge. Here, we assume that the dark current is the major contributor to the shot noise, thus deduce to the equation (3) [7]. This simplification has been used to evaluate the photoresponse of 2D layered materials, like graphene [23] and WSe_2_ [24].

As can be seen from Fig. 2c, the device has a very stable and repeatable photoresponse to 1550 nm light after several cycles. Its response time is extremely fast and could reach 30 ms in rise and 60 ms in decay, respectively. This is due to the fact that as-grown ultrathin Bi_2_O_2_Se nanofilms have no surface trap states and shallow defect energy levels. Finally, as shown in Fig. 2d, *I*_*ph*_ increases monotonically with P increasing, following a relationship *I~P*^*α*^. Here, α is deduced to be 0.99 for Bi_2_O_2_Se by fitting the experimental data, suggesting that the photocurrent is mainly determined by the amounts of absorbed photon [7]. The photoresponsivity of multilayer Bi_2_O_2_Se–based photodetectors is around 68 A/W, which exhibits an extremely high performance as a photodetector.

**Fig. 3:**
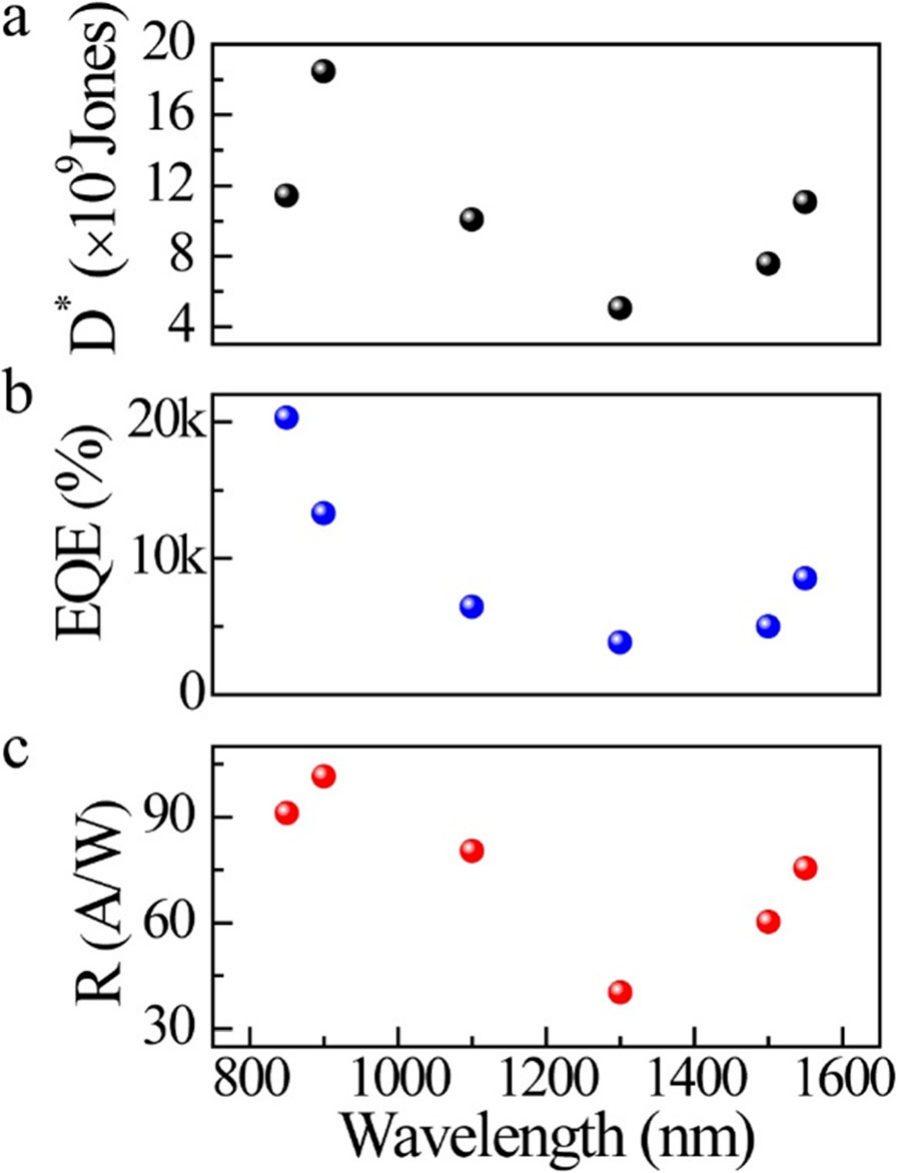
NIR photoelectric performance of multilayer Bi_2_O_2_Se–based photodetector. **a** Photoresponsivity, **b** external quantum efficiency, and **c** detectivity as a function of NIR wavelengths

Next, the photoelectric response performance of multilayer Bi_2_O_2_Se–based photodetector to the NIR wavelengths (850–1550 nm) was systematically studied. According to the calculation by stated formulas (1)–(3), the photoresponsivity, external quantum efficiency and detectivity are demonstrated in Fig. 3. It can be found that the device has a very high photoresponsivity to the NIR band, which reaches 101A/W (900 nm). In addition, multilayer Bi_2_O_2_Se–based photodetector owns an ultrahigh *η*, which exceeds 20,000% at 850 nm, indicating its excellent photoelectric conversion capability. Its detection rate can reach 1.9 × 10^10^ at 900 nm, showing a perfect signal-to-noise ratio as a photodetector. In our measurement, the dark current of the device always maintains at a relatively stable value (0.5 μA). Therefore, the trend of D* (as a function of incident wavelength) is similar to the trend of *R*. Obviously, compared with thin-layer Bi_2_O_2_Se reported by Ref. [7] and Ref. [8], multilayer Bi_2_O_2_Se has higher photoelectric responsivity and external quantum efficiency (source-drain bias voltage, 1 V, which is the same as Ref. [7] and Ref. [8]), while still keeps a relatively fast response time and high detection rate. Noticeably, Ref. [8] only reported the intrinsic response time (1 ps) of material by pump–probe technique but with no device response time of Bi_2_O_2_Se photodetectors [8].

In general, 2D layered materials have not yet shown such high sensitivity in NIR detection range. For example, transition metal dichalcogenides (TMDs) usually have too large band gaps to detect IR light [17], while for graphene, it shows high-speed photoresponse but very low intrinsic sensitivity less than tens of mA/W [25]. Although the photoresponsivity can be improved by fabricating atomically heterostructures [26–28], it still does not perform perfectly in the NIR detection. Compared with other 2D materials (Table 1), multilayer Bi_2_O_2_Se–based photodetector shows a more excellent photoelectric performance, especially a high *R* and a high *η*. Noticeably, if the chemical etch was applied to optimize the geometry of multilayer Bi_2_O_2_Se nanofilms [15], the performance of the device may be further enhanced.

**Table 1:** Comparison of the performance of different room temperature NIR photodetectors. *W* represents response wavelength, *R* represents the photoresponsivity, *η* represents the external quantum efficiency, D^*^ represents detectivity, and *t* represents response time

	*W* (nm)	*R* (A/W)	*η* (%)	D^*^ (Jones)	*t*	Reference
Multilayer Bi_2_O_2_Se	850–1550	101	20,300	1.9 × 10^10^	< 30 ms	This work
Thin-layer Bi_2_O_2_Se	808	6.5	999	8.3 × 10^11^	2.8 ms	7
Thin-layer Bi_2_O_2_Se	300–1700	65	——	3.0 × 10^9^	1 ps (intrinsic)	8
Graphene	1550	0.5 × 10^−3^	16	——	< 25 ps	25
MoTe_2_-MoS_2_	550–1550	0.046	——	——	25 s	26
GO-GNR	1550	1	80	——	2 s	27
MoS_2_/b-P	532–1550	22.3	5000	3.1 × 10^11^	70 μs	28

**Fig. 4:**
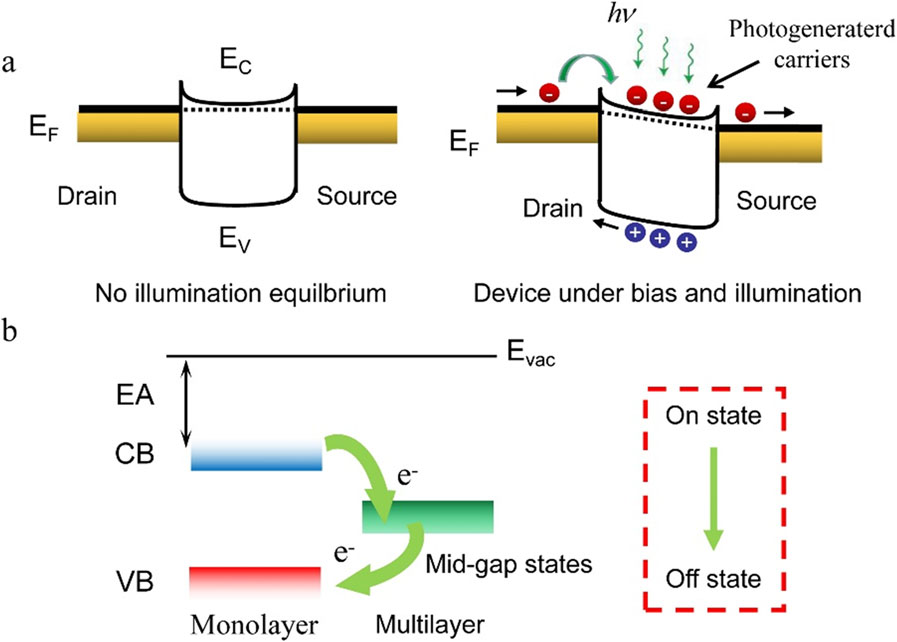
Physical mechanism of multilayer Bi_2_O_2_Se–based photodetector. **a** The behavior of photo-generated charge carriers of multilayer Bi_2_O_2_Se-based photodetector. Here, E_F_ is the Fermi level energy, E_C_ is the minimum conduction band, E_V_ is the maximum valence band. **b** The recombination process (“on” state to “off state”) of Bi_2_O_2_Se-based photodetector. Here, CB is conduction band, CV is valence band, E_vac_ is vacuum energy, EA is electronic affinity

The photo-response physical process of Bi_2_O_2_Se-based photodetectors can be explained by a simple energy band diagram (Fig. 4a). With no illumination and without applying drain bias, the device is in its equilibrium state and with no current flow in the channel. Illuminating the device with NIR light will result in light absorption and excitation of electron-hole pairs, which can be extracted by applying a drain-source bias [29–31]. Since the Schottky barrier in Bi_2_O_2_Se-metal contact is very low, the photo-generated charge carriers could easily pass the barrier [16–18]. Therefore, multilayer Bi_2_O_2_Se–based photodetector exhibited an excellent photoelectric performance.

Importantly, the mechanism of enhanced photoelectric performance should be discussed. Theoretically, the optical adsorption of multilayer Bi_2_O_2_Se is higher than that in thin-layer Bi_2_O_2_Se, which can induce higher photocurrents *I*_*ph*_ [14, 20]. The incident power *P(x)* as a function of distance *x* could be expressed as *P(x) = P*_*in*_*·e*^*−α·x*^, where *α* is the absorption coefficient of the Bi_2_O_2_Se nanofilms at the incident photon energy. The amount of power absorbed by a slab of Bi_2_O_2_Se with thickness*Δx* at a distance *x* from the surface is *dR*_*a*_ *= − (dP/dx)·Δx*. Then, the total power absorbed by the Bi_2_O_2_Se film of thickness *d* is *R*_*a*_ *= P*_*in*_·(1 − *e*^*−α·d*^). For *α·d* << 1, the absorbed power can be written as *R*_*a*_ *= P*_*in*_*·α·d* [16, 19]. Here, the thickness *d* of in our experiment is 5 times and 3 times of Ref. [7] and Ref. [8], respectively. As a matter of fact, multilayer Bi_2_O_2_Se nanofilms in our work would have a better *R*. However, though with the increase of optical adsorption, multilayer Bi_2_O_2_Se have some drawbacks, such as a higher density of states (DOS), thus causing more mid-gap states compared with monolayer (or thin-layer) [13, 14]. As Fig. 4b shows, when the device turns from “on state” to “off state,” the excited electrons in higher bands of multilayer Bi_2_O_2_Se will firstly transit to the mid-gap states, and then return to ground band [16, 17, 19]. In other words, the carrier lifetime *τ* will inevitably incline. The same situation will emerge when the device turns from “off state” to “on state.” Interestingly, compared with previous work, multilayer Bi_2_O_2_Se nanofilms still have a fast response time, which is satisfied in many applications [1–3]. This means the existence of mid-gap states may not be detrimental to the dynamic performance of Bi_2_O_2_Se nanofilms. Last, for greatly enhanced *η*, two main reasons play pivotal roles. Firstly, the increased layers improve the absorbance of incident photons. In addition, the existence of mid-gap states allows for more transition channels for excited electrons. Thus, *η* increases significantly [16, 19].

## Conclusions

In summary, we have presented the photoelectric properties of multilayer Bi_2_O_2_Se (thickness ~ 30 nm)–based photodetector. Multilayer Bi_2_O_2_Se demonstrates an ultra-sensitive photoresponse from 850 to 1550 nm with good reproducibility at room temperature, including a high photoresponsivity, a quick response time, a high external quantum efficiency, and a high detection rate. Results indicate that multilayer Bi_2_O_2_Se has a relatively better photoresponse than that of thin-layer.

## Data Availability

All data are fully available without restriction.

## Abbreviations

NIR: Near-infrared
IR: Infrared
TFT: Thin-film transistor
AFM: Atomic force microscope
SEM: Scanning electron microscope
CVD: chemical vapor deposition
TMDs: Transition metal dichalcogenides
